# Ontology for Semantic Data Integration in the Domain of IT Benchmarking

**DOI:** 10.1007/s13740-017-0084-9

**Published:** 2017-11-13

**Authors:** Matthias Pfaff, Stefan Neubig, Helmut Krcmar

**Affiliations:** 1 fortiss GmbH, An-Institut Technische Universität München (TUM), Munich, Germany; 2 Technische Universität München (TUM), Munich, Germany

**Keywords:** Ontology, Domain modeling, Information systems, IT benchmarking, Knowledge representation, Semantic data

## Abstract

A domain-specific ontology for IT benchmarking has been developed to bridge the gap between a systematic characterization of IT services and their data-based valuation. Since information is generally collected during a benchmark exercise using questionnaires on a broad range of topics, such as employee costs, software licensing costs, and quantities of hardware, it is commonly stored as natural language text; thus, this information is stored in an intrinsically unstructured form. Although these data form the basis for identifying potentials for IT cost reductions, neither a uniform description of any measured parameters nor the relationship between such parameters exists. Hence, this work proposes an ontology for the domain of IT benchmarking, available at https://w3id.org/bmontology. The design of this ontology is based on requirements mainly elicited from a domain analysis, which considers analyzing documents and interviews with representatives from Small- and Medium-Sized Enterprises and Information and Communications Technology companies over the last eight years. The development of the ontology and its main concepts is described in detail (i.e., the conceptualization of benchmarking events, questionnaires, IT services, indicators and their values) together with its alignment with the DOLCE-UltraLite foundational ontology.

## Introduction

IT benchmarking is based on the insight that by observing organizations and analyzing their performance, an organization can transform the way it conducts business [4]. Such a transformation is usually achieved by applying lessons learned from benchmarking results to their own organization [5, 40]. Information is generally collected during a benchmark exercise using questionnaires on a broad range of topics, such as employee costs, software licensing costs, and quantities of hardware or software. Moreover, there are different types of benchmarks that generally focus on the same subject from different points of view, especially in the domain of IT benchmarking. Although the different benchmark types measure the same object from different perspectives, a direct link is often difficult to establish between these collected data. Research in the field of IT benchmarking typically focuses on structuring, standardizing and generalizing IT service catalogs and their implementation within companies [11, 32] to model internally provided IT services in a standardized manner. Because IT service catalogs are commonly designed for internal or individual purposes only, they are often not directly comparable, especially when attempting to compare the across organizational boundaries. This is because the concept of a uniform data description and data management is not considered even though it is strongly recommended for such measurement problems in the domain of IT benchmarking [41, 54].

Currently, the number of studies in the IS literature addressing these data integration challenges across different types of IT benchmarks is limited and most literature sources omit facts related to the data quality, the data integration and the comparability of different types of benchmarks. This is because of the lack of a uniform description of any arbitrary performance parameter and key performance indicator (KPI) that is measured during a benchmark and because of the lack of a uniform description of the relationships between these parameters [42] relevant for comparability. However, a domain-specific ontology may represent a solution to ensure that the collected data are meaningful and to overcome these limitations of data comparability [28, 51]. Similar ontology-based approaches for enhancing the data quality have been successfully implemented in related fields of research, for example, for linking IT infrastructure and business elements (cf. [2]).

Since there are numerous challenges related to data integration specific to not only the domain of IT benchmarking but also related fields, such as IT service management (ITSM), in this work, we describe an IT benchmarking ontology, an ontological formalization of all relevant elements, attributes, and properties in this domain, following the description logic fragment of the Web Ontology Language (OWL) 2 language [35]. Thus, this work contributes to the data comparability problem because of the lack of standardization by showing to which degree of abstraction the conceptualization of relevant concepts needs to be covered by an ontology in the domain of IT benchmarking and what basic relationships need to be modeled within the core IT benchmarking ontology (ITBM). While the ITBM ontology provides the common understanding of concepts and relations within the domain of IT benchmarking the semantic foundation is achieved by grounding the ITBM ontology in an upper ontology, a ”foundational ontology.” For this reason, the ITBM ontology is linked to Dolce UltraLite (DUL) [15]. Grounding in a foundational ontology ensures the semantic interoperability of distinct conceptualizations from different (domain) ontologies [24].

The paper is organized as follows: Sect. 2 provides an overview of the relevant literature on IT benchmarking/service management, foundational ontologies, and ontologies in related domains. The methodology for the development of the ITBM ontology is described in Sect. 3. Section 4 introduces the proposed ITBM ontology and gives an overview of the document structure used to build the domain ontology. Section 5 outlines the application and use case of the ITBM ontology. Finally, Sect. 6 provides the conclusion and perspectives for future in terms of ontology extension.

## Background

### The Domain of IT Benchmarking

As a systematic process for improving organizational performance, benchmarks can be classified according to the type of study (e.g., processes, products, strategies or generic objects) (cf. [6]). Benchmarking partners may be units of the same organization, competitors in the same or different geographical markets or organizations in related or unrelated industries. Thus, a distinction is drawn between internal and external comparisons of these performance measurements. Whereas an internal performance measurement focuses on the operation of a single company, an external performance measurement focuses on different companies. An overview of the different types of benchmarks is presented in Table 1.

**Table 1:** Types of Benchmarks (based on [6])

Type	Description
Process Benchmark	Compares operations, work practices or business processes
Product Benchmark	Compares products or services
Strategic Benchmark	Compares organizational structures, management practices and business strategies
Internal Benchmark	Compares products or services of business units within a single organization
Competitive Benchmark	Compares performance with a direct competitor. The object of investigation may include products, services, personnel policies, etc.
Functional Benchmark	Compares one or more non-competitive organizations in terms of particular business functions or processes
Generic Benchmark	Compares an organization or business unit with the best-performing organization, irrespective of the type of industry

**Fig. 1:**
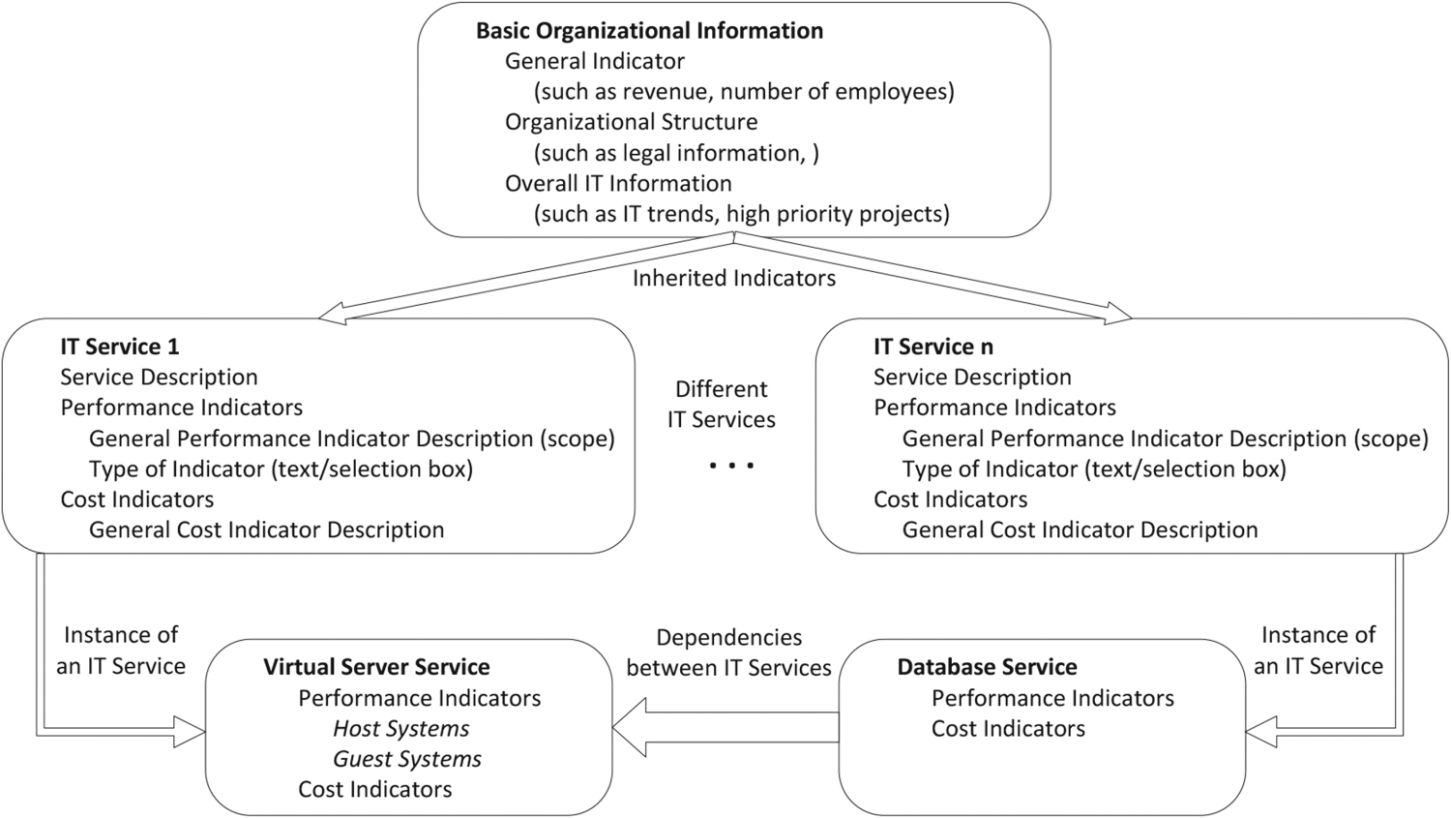
Structural overview of the IT service catalogs used to build the ontology. Services are segmented first (e.g., cost or performance indicator) and optionally further split into indicator groups (e.g., host systems). Services may include the costs of other services (e.g., a database service includes the cost also specified in a virtual server service) (based on Pfaff and Krcmar [43])

A benchmark can be subdivided into several process phases, beginning with the initial conception which describes the object of investigation and ending with optimizing and re-organizing internal (business) processes. In each of these phases of a benchmark numerous data (KPIs) are collected in various data formats or data structures. These data consist of both qualitative and quantitative statements and are (recurrently) collected through the entire benchmarking cycle for every benchmark. Furthermore, they are collected for every benchmarking participant. In IT benchmarking, the scope of the collected data is generally limited to IT-related performance indicators, regardless of whether they were collected within a strategic or generic benchmark. Thus, these data (indicators) are similar in a semantic manner, as they are related to specific IT aspects, even if acquired within different types of benchmarks. More generally, different IT benchmarks often measure the same IT objectives from different vantage points. Therefore, such collected data are semantically related to each other for this specific objective which was measured within different benchmarks.

The structural layout of an IT service catalogs can be generalized to (i) basic organizational information (such as the number of employees or revenue), subsequently referred to as basic data services, and (ii) 20 additional IT services, describing more specific aspects of IT offerings (cf. Fig. 1). These IT services provide some general information about what the service offering is about (for example, providing a mailbox or a virtual machine/server) and detailed information about performance and cost indicators that are used to measure the performance of this service. Note that calculations of indicators may be dependent on different services. For example, a storage service contains all costs associated with disk storage in a data center; however, some of those storage-specific costs are also required within a more general IT service such as in the context of server costs (as disk storage is associated with servers in general). Additionally, costs originally related to the database service are based on both the general server costs as part of the infrastructure component and the more specific disk storage costs. Again, some cost indicators of the database service depend on the performance indicators of the server and data storage service. It is also possible that IT services could inherit indicators or values from the basic organizational information (such as the total number of employees of an organization) to perform further calculations within a specific service based on such a basic indicator. Figure 1 shows the structural layout of the IT service catalogs and IT service descriptions used to build the ontology.

In short, IT services are mono-hierarchically structured. Each top-level service consists of a set of subordinated service segments and, optionally, additional indicator groups. As shown in Fig. 1, the basic data service’s segments correspond to general organizational information (e.g., organizational structure and IT costs), and the remaining IT services are segmented by whether they are cost or performance indicators and, optionally, grouped into smaller logical units (for example, the host or guest systems in the context of the virtual server service).

### Foundational Ontologies and Ontologies in Related Domains of ITBM

To link data (bases), that are similar in a semantic manner, the use of ontologies has become popular in recent years, with a particular focus on the representation of business processes (cf. [17, 50] or for the purpose of enterprise modeling (cf. [52]). By nature, when an ontology is built with a focus on business processes or enterprise modeling, it lacks the information needed to shift the focus to financial aspects, which are of crucial importance in the domain of IT benchmarking. Although such ontologies, such as the Edinburgh Enterprise Ontology (EEO) by Uschold [52], the TOronto Virtual Enterprise (TOVE) by Fox and Grüninger [12] and the Design and Engineering Methodology for Organizations (DEMO) by Dietz and Hoogervorst [9], are used for enterprise modeling, they differ in the meaning of key terms, as they are not grounded in a foundational ontology. Further, aside from the lack of a shared understanding of equal concepts in these ontologies, they do not address IT infrastructure and IT costs nor do they focus on IT-comparable IT services in general across company boundaries, which is crucial for the domain of IT benchmarking. This situation holds true for ontologies in the context of ITSM (cf. [13, 53]), for ontologies and IT governance frameworks in the context of the Information Technology Infrastructure Library (ITIL) [38] and for related ontologies such as the GoodRelations ontology [26] and the Financial Industry Business Ontology (FIBO) [8]. Whereas the business model ontology (BMO) [39] only focuses on the conceptualization of economic aspects within a single enterprise, the e^3^-value ontology [21] only focuses on the conceptualization of economic aspects within a network of enterprises. Other, more domain-specific ontologies focus on the modeling of the aspects of an enterprise’s accounting aspects, such as the resource-agent-event (REA) ontology [18], which is used to define the architecture of an accounting information system (AIS). Since the REA ontology is not grounded in a foundational ontology, it is unclear what is meant by an economic event.

One initial approach for measuring the impact of IT infrastructure changes on business processes and vice versa by an ontology was introduced by vom Brocke et al. [2]. The focus of this study is the linkage of (inner) organizational process levels to their IT-resource level. However, to (semi-) automatically compare IT-related and business-related performance indicators across organizational boundaries, a more fine-grained conceptualization of such information is needed. Especially if the ontology is directly used to link and access external data sources (i.e., directly map ontology concepts to IT business-related KPIs) to analyze the organizational performance of (IT) services, the conceptualization needs to be closer to the structure of IT service catalogs than to the abstract description of organizational processes or IT resources.

As previously stated, upper ontologies, or ”foundational ontologies,” are used to ensure the semantic interoperability of distinct conceptualizations from different domains [24]. Thus, several of these foundational ontologies have been recently developed. The Suggested Upper Merged Ontology (SUMO) [36], the Basic Formal Ontology (BFO) [48], the Descriptive Ontology for Linguistic and Cognitive Engineering (DOLCE) [16], the Unified Foundational Ontology (UFO) initially presented by Guizzardi and Wagner [25], and the General Formal Ontology (GFO) proposed by Herre [27] are some prominent examples of this type of ontology. The BFO was developed for the support of information retrieval, analysis and integration in scientific and other domains. It was developed to be very generic and to incorporate both three-dimensionalist and four-dimensionalist perspectives on reality. In contrast to BFO, DOLCE captures ontological categories underlying natural language and human commonsense [33]. As a descriptive ontology, DOLCE distinguishes between things and events, which correspond to organizations (things) and benchmarks (events) in the domain of ITBM. In DOLCE, the differences between these entities are related to their behavior in time, and they are linked by participation relations (similar to a participation within a benchmark), whereas in BFO (as a realist ontology) such branches are completely independent of each other. Thus, DOLCE offers a better support for representing temporal qualities (e.g., a benchmark as a time-specific event) and properties (e.g., a specific type of benchmark) and values (e.g., a particular benchmark of a specific type). Since a lightweight version of DOLCE is provided with DUL [15], being sufficient in terms of expressiveness and complexity, DUL was used for grounding the ITBM ontology. Note that for grounding the ITBM ontology in a foundational ontology, GFO and SUMO would also have been appropriate, as they also provide sufficient temporal conceptualizations. However, since no lightweight version of GFO exists and since the extensive and detailed taxonomy of SUMO is not needed, the ITBM ontology is grounded in DUL to provide a lightweight solution. In contrast to the previously mentioned foundational ontologies, which are based on OWL, UFO is based upon OntoUML [24]. As a result, and since the ITBM ontology was implemented in OWL, UFO and its extensions (UFO-A, UFO-B, UFO-C and UFO-S) were not considered further in the investigation. OWL was chosen for the development of the ITBM ontology to ensure further linkage possibilities to the previously mentioned domain ontologies (such as FIBO and BMO).

## Methodology

For the development of the ITBM ontology, we implemented a customized process based on the NeOn framework for ontology engineering [49]. NeOn offers nine different scenarios consisting of 59 activities. The basic activities for each ontology development process are bundled in the NeOn core scenario. To perform a certain scenario, the scenario is mapped to the phases of an underlying life cycle model. Two life cycle models are supported; a waterfall model with a variable number of phases (depending on the scenario to be performed) and an iterative and incremental model. The iterative and incremental model is a sequence of subsequently performed waterfall models (i.e., iterations), each of which may be based on a different scenario; the chosen scenario defines the different phases to be performed during a specific iteration. Activities are described in a glossary of terms, aiming to give commonly accepted definitions for certain activities. Most activities come with a set of comprehensive descriptions consisting of functional descriptions (e.g., definition, goals, and input/output).

The IT benchmarking ontology as presented in this work is the result of a number of iterations of the overall ontology engineering process, which is based on an iterative and incremental life cycle model. So far, both the NeOn core scenario and the NeOn scenario for the reuse of ontological resources have been used. In addition to this customization, we further adapted some of the NeOn activities to fit our needs therein keeping the engineering process as lightweight as possible. In the following, subsequently performed activities are described in more detail in the order or their execution.

- Knowledge Acquisition. According to the NeOn specification for the knowledge acquisition process, three different activities were performed: (i) ontology elicitation to acquire conceptual structures and their instances by domain experts; (ii) ontology learning to (semi-)automatically transform unstructured, semi-structured and structured data sources into conceptual structures; and (iii) ontology population to (semi-)automatically transform unstructured, semi-structured and structured data sources into instance data. Within the IT benchmarking ontology engineering process, the ontology population activity is not performed during the ontology design phase, as the IT benchmarking ontology solely contains conceptual knowledge. Analogously, knowledge elicitation is limited to gathering conceptual knowledge. Ontology learning was conducted to support the domain experts in performing the ontology elicitation activity; here, existing service catalogs and databases were analyzed using Natural Language Processing (NLP) techniques to extract the most important concepts, as described in detail in [42].
- Ontology Requirements Specification. The main challenge during the specification activity was to identify a set of appropriate competency questions (CQs) to describe the requirements to be fulfilled by the final ontology as the ontology is used for accessing external data sources. Thus, the CQs are questions the ontology should be capable of answering, based on the results of the external attached data sources. Following the NeOn guidelines, Table 2 shows the categorized and prioritized CQs for the ITBM ontology and the corresponding query-style answers.
- Ontology Conceptualization. To organize data and information according to the specified requirements in the domain of IT benchmarking, we created a conceptual domain representation as proposed by NeOn, which was stepwise refined. Starting with a list of terms obtained from the ontology requirements (i.e., extracted from the CQs) and deriving concepts from those terms, we enhanced this domain representation until reaching a semi-formal, graphical model of the intended ontology. Moreover, to enhance the general quality of the final model and to specify concepts in more detail, we used existing data sources (such as service catalogs and related databases (cf. Sect. 4) for the conceptualization, and additionally utilized the NeOn framework for ontology engineering [49]).
- Ontology Reuse and Aligning. Existing (non-)ontological resources are used for the development of the ITBM ontology. These resources encompass ITBM data collected over the last eight years in the context of research activities on ITBM at the research institute fortiss and the Technische Universität München (TUM). Moreover, existing domain ontologies in related domains are identified and evaluated for their suitability in the context of ITBM (for additional details see Sect. 2.2). By grounding the ITBM ontology in the upper ontology DUL, the semantic foundation of the ITBM ontology is achieved. To achieve this, relevant concepts in DUL and the ITBM are identified and linked (see Sect. 4).
- Ontology Implementation. Within the scope of ontology implementation, the conceptual model obtained during the conceptualization activity is implemented using OWL 2 DL [35]. Note that the expressiveness of OWL 2 entailment is required to formally represent more complex properties, especially property chains, that is, inferring a new property between two concepts based on a chain of existing properties already linking them (complex role inclusion) [22]. With regard to the huge number of indicators, the implementation process is supported by (semi-)automatic tools (i.e., a software script) that generate concepts of the ontology from previously extracted term lists derived from the existing databases.
- Ontology Annotation. To keep the ontology readable for humans, we conduct an activity for annotating the ontology. In addition to general information (e.g., the ontology version), concepts and properties are annotated using *rdfs:label* and *rdfs:comment*. In the same way as the implementation activity, this activity is (semi-)automatically supported by the use of existing databases in this domain.
- Ontology Evaluation. Before the ontology is published, ontology evaluation is performed. Here, the final ontology is first evaluated against the CQs listed during the specification activity. Then, different tools (i.e., the HermiT reasoner [19] and the OntOlogy Pitfall Scanner (OOPS) [44]) are applied to ensure both that the ontology is consistent as well as its general quality.

**Table 2:** Extract of competency questions created during the *Specification* activity, grouped by pre-established categories as suggested by NeOn: (i) Indicator Structure, (ii) Individual Benchmarks and (iii) Participants and Values. Square brackets indicate lists of values

Group	Competency Question (CQ1-CQ20)	Exemplary Answer
Indicator	What performance indicators do exist?	[NumberOfUsers]
Structure (CQ1-CQ6)	What performance indicators are contained in the BENCHMARK_NAME in YEAR?	[NumberOfUsers]
	Regarding BENCHMARK_NAME of YEAR, how many cost indicators have been answered by all participants?	NUMBER
	What IT services are of interest (i.e., have had values provided for) for the ORGANIZATION_NAME ?	[BasicDataIndicator]
	How frequent is the revenue indicator queried within the existing benchmarks?	NUMBER
	How many values have been provided for the revenue indicator of the SERVICE_NAME in total?	NUMBER
Individual	How many benchmarks exist?	NUMBER
Benchmarks (CQ7-CQ11)	In which years was the BENCHMARK_NAME conducted?	[YEAR]
	Which indicators have been queried in at least two benchmarks?	[HardwareCost]
	How many values have been provided for the number of employees indicator in total?	NUMBER
	Which organizations have participated in which benchmarks?	[(ORGANIZATION_NAME, BENCHMARK_NAME, YEAR)]
Participants	How many organizations do exist?	NUMBER
and Values (CQ12-CQ20)	How many organizations have participated in at least one benchmark?	NUMBER
	Does ORGANIZATION_NAME participate in at least one benchmark called BENCHMARK_NAME ?	YES/NO
	What is the yearly revenue of ORGANIZATION_NAME ?	[(YEAR, NUMBER)]
	What was the average hardware costs for BlackBerry devices in YEAR?	NUMBER
	What was the greatest value of hardware costs for BlackBerry devices provided in YEAR?	NUMBER
	What are the hardware cost for BlackBerry devices in YEAR by ORGANIZATION_NAME?	[(ORGANIZATION_NAME, NUMBER)]
	Regarding YEAR, what was the average number of employees of all organizations having a revenue between $NUMBER_1 and $NUMBER_2 ?	NUMBER
	Regarding YEAR, what was the minimum number of employees of organizations having a revenue between $NUMBER_1 and $NUMBER_2 ?	NUMBER

In addition to the subsequent activities as described above, the IT benchmarking ontology engineering process is supported by a number of side activities as also suggested by NeOn. Those activities are described as follows:

- Ontology Quality Assurance and Control. The control activity refers to process monitoring and ensures that the subsequent activities described above are performed and completed correctly. The ontology quality assurance activity ensures the quality of the ontology implementation process and its artifacts. During the development of the IT benchmarking ontology, the process was monitored and controlled constantly using checklists.
- Ontology Documentation. While developing the IT benchmarking ontology, the utilized and created documents and artifacts (e.g., including reasoning of design decisions and code fragments) were collected and ordered for documentation purposes.

As stated before, to allow the ITBM ontology to be machine-processable, it is implemented in OWL (more specifically, following the OWL 2 DL fragment [35]), a World Wide Web Consortium (W3C) standard [3, 34]. Thus, the OWL ontology consists of the following: (i) classes as sets of individuals, (ii) individuals as instances of classes (i.e., real-world objects in the domain) and (iii) properties as binary relations between individuals. In addition to the implementation of the domain knowledge, it is possible to define cardinality ranges and other constructs (e.g., taxonomies) allowing inference within an ontology. Moreover, a reasoning engine was used during the development process to avoid inconsistencies in the specifications of the ontology classes and properties. The corresponding ITBM ontology was modeled using the open-source ontology editor Protégé [45], as it is one of the most common tools for ontology development [30].

**Fig. 2:**
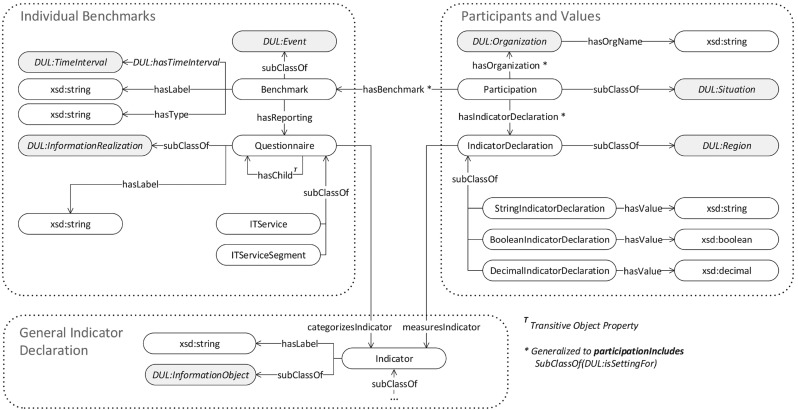
IT benchmarking ontology consisting of three different sections: (i) Individual Benchmarks, (ii) Participants and Values and (iii) General Indicator Declaration. Solid arrows indicate data or object properties, with their direction being defined by *rdfs:domain* and *rdfs:range* [1]

## IT Benchmarking Ontology

The IT benchmarking ontology was initially built based on already-existing IT service descriptions and catalogs of numerous small- to medium-sized enterprises and several questionnaires from different IT benchmarking approaches. As previously stated, these data were collected over the last eight years in the context of research activities and were supervised and evaluated within different benchmarking approaches (cf. [42, 47]). These data encompass results from strategic and consortial IT benchmarks. Subsequently, as a result of the different data acquisition channels of online web platforms, Excel questionnaires and other sources (cf. [10, 55]), different distributed data sources were used to derive the concepts of the ITBM ontology. The database consists of 1007 unique descriptions of key performance indicators, which are composed of 25 service catalogs from individual companies. In addition, the underlying data for the ontology development consist of 708 data sets from consortial IT benchmarks. These data sets encompass questions on 15 IT services answered for 10 companies as an yearly average over the last six years. Furthermore, IT benchmarking results from 112 different companies were used to extend the database for the ontology development. These data were acquired over the last eight years within a strategic benchmark based on [46], and each data set consists of 1,612 quantitative and qualitative data points of a single organization. As previously stated, the existing service catalogs and databases were analyzed using NLP techniques to extract the most important concepts and terms relevant to building the ontology (for more details on NLP, see Pfaff and Krcmar [42]).

As described before, the ontology was implemented following the OWL 2 DL fragment [35] and using the common vocabularies based on ITIL [38]. Moreover, the alignment to DUL [15] was added to make the ontological commitments explicit and to specify the intended meaning of the introduced concepts [23].

### Top-Level Description

Starting with the top-level description of the proposed benchmarking ontology, the ontology can be divided into the following three sections: *Individual Benchmarks* (equivalent to one specific benchmark), *Participants and Values* and the *General Indicator Declaration*. *Individual Benchmarks* section introduces concepts to describe, processes relying on different IT service descriptions or questionnaires, including a customizable structure of selectable indicators (measured within a benchmark). Participants (viz. organizations) and their values, which may be instantiated based on these concepts, are described in *Participants and Values* section. The indicators themselves and their hierarchical and intermediate relationships are organized in a three-layer taxonomy referred to as the *General Indicator Declaration* section. The *General Indicator Declaration* is described in more detail in Sect. 4.2 because of its complexity. Figure 2 provides a conceptual overview of the three ontology sections and the relations in between. Gray nodes indicate inheritances from DUL concepts and properties. The nodes of the graph illustrated in Fig. 2 refer to *concepts* (i.e., classes) or *datatypes* [35] of the ontology, whereas the edges refer to *properties* provided by the ontology.

#### Individual Benchmarks

An IT benchmark is identified by a specific name. As described in Sect. 2, a benchmark may be conducted once or several times within various time periods. In the following, an *individual benchmark* refers to a single conduction of a benchmark that a company is participating in (i.e., an instantiation of the Benchmark class), whereas the benchmarking specification in general refers to a concept of a benchmark that is performed numerous times in different capture or time periods. In other words, two individual benchmarks can be conducted based on two different indicator structures and indicators, or these individual benchmarks can differ in the year of being conducted. In both cases, these benchmarks are represented as a delimited instance within the ontology to uniquely identify individual benchmarks.

As already mentioned in Sect. 2, indicators may be captured in different contexts. For example, whereas an individual benchmark may be based on specific questionnaires (i.e., indicators are grouped in arbitrary categories), the indicator structure of another benchmark may be completely based on a traditional service catalog (i.e., indicators are grouped by the IT service that they belong to). To represent and distinguish the contexts a specific indicator is captured within individual benchmarks; different concepts have been introduced to represent an indicator structure (i.e., *Questionnaire*, *ITService* and *ITServiceSegment*).

In the following, the concepts that an *Individual Benchmark* consists of are described in more detail:

- Benchmark. A benchmark can be seen as a time-specific event for the conduction of a benchmark. Thus, the Benchmark class is grounded in the *DUL:Event* concept. An instance must have at least one label, containing the benchmark’s name, a type and its specific time interval of conduction. Such a *TimeInterval* is defined for events within DUL and may be freely specified by utilizing the *DUL:hasTimeInterval* property. The *hasType* property refers to the set of benchmark types as described in Table 1 and is therefore limited to those values. Each benchmark has to be assigned to one or two of these benchmarking types. The labels of a benchmark are represented by arbitrary strings, referring to benchmark names, for example expressed in one or multiple languages. For connecting to DOLCE, both *hasLabel* and *hasType* have been defined as a sub-property of *DUL:hasDataValue*.
- Questionnaire. During a benchmark event, indicator values are reported by utilizing exactly one previously specified questionnaire that defines a structure for capturing these data of the KPIs. These questionnaires are connected to a benchmark instance using the *hasReporting* property. Within the ITBM ontology, a questionnaire refers to a physical object (e.g., paper sheets), is grounded in *DUL:InformationRealization* and is labeled by at least one headline (e.g., multiple headlines for multiple languages). Indicators are more abstract information objects and are linked to a questionnaire using the *categorizesIndicator* property, which is a sub-property of *DUL:realizes*. A questionnaire or a group of questionnaires consists of different indicators focusing on different aspects or activities within an IT department, such as general service offerings or more generic questions. For more details on the structure of a non-service-based ITBM see Riempp et al. [46]. A questionnaire can be further nested into sub-questionnaires coupling questions to a specific topic of interest to compare through the benchmark. This results in a mono-hierarchical structure that can be realized using the transitive *hasChild* property, which is a sub-property of *DUL:hasPart* and defines a questionnaire to be a part of another questionnaire.
- ITService. An IT service consists of a set of different activities to be performed by an IT department to meet specific business or IT demands. Thus, as the structure of an individual benchmark is based on IT service catalogs, describing the parts of this service in natural language and based on indicators for the measurement of the service KPIs, this structural information is represented by the ITService concept. In other words, an ITService is a specialization of the more general questionnaire consisting of KPIs that are directly linked to IT service activities and their organizational resources (such as costs or human resource). Once an IT service is defined, it can also be further divided into sub-services.
- ServiceSegment. It is also possible to structure an IT service in more fine-grained ways. Thus, an IT service can be divided into a set of smaller service segments. For example, an indicator set of a service could be divided into indicators referring to mobile and stationary IT systems in accordance with the description of the underlying IT service catalog structure. Moreover, a service segment may be further divided into smaller segments if necessary to maintain the structural information of this service.

#### Participants and Values

In the domain of IT benchmarking, a participant represents an organization contributing values of benchmarking indicators (answering questions) specific to an individual benchmark. In the ontology, this organization is represented as a class (i.e., Organization) and connected to an individual benchmark (i.e., Benchmark). The contributed values are indicated by the use of the Participation and IndicatorDeclaration classes. The description of these classes is as follows:

- Organization. A participant represents an organization participating in specific benchmarks (minimum of one) and is identified by its name. To foster reuse, it refers to the *DUL:Organization* concept provided by the DUL ontology.
- Participation. According to the IT benchmarking process an organization contributes its KPIs (alues) while participating in a specific benchmark. In DUL, such participations are usually represented by *DUL:involvesAgent* and the *DUL:isAgentInvolvedIn* properties, established between an event and its participants. However, this approach is insufficient, as a single property cannot represent the ternary relation of a benchmark and the participant in combination with the contributed values (cf. [37]). Therefore, the participation has been implemented based on the Nary Participation ontology design pattern [14], which specifies a reified participation concept and a *participationIncludes* property to link participation with (i) at least one event (e.g., the benchmark), (ii) at least one object (e.g., the participant and its values) and (iii) at least one time interval to describe when the participation in the event occurred. Regarding the ITBM ontology, however, the time index of the participation (iii) was removed as we are only interested in the time span for which collected values are valid (i.e., given by the benchmark event) rather than the time span in which values were collected. Moreover, to further specify the role of a certain entity during one participation, additional properties (i.e., *hasBenchmark*, *hasOrganization* and *hasIndicatorDeclaration*) inheriting from *participationIncludes* have been introduced.
- IndicatorDeclaration. For each indicator value, provided by a specific organization, an *IndicatorDeclaration* (grounded in the *DUL:Region* concept) is instantiated. This is included in one participation and represents the measures of exactly one specific indicator. An IndicatorDeclaration has one or multiple values attached to it. Currently, these values can be in the format of strings, Booleans or decimals, represented by the corresponding subclasses. For each pair consisting of a participation and an indicator, only one IndicatorDeclaration is instantiated. Thus, using a subclass referring to a specific unit type, instead of the more abstract IndicatorDeclaration, an indicator can only be described by a single type of unit at one time, even if more values are attached to it (e.g., a list of values).
- StringIndicatorDeclaration. A *StringIndicatorDeclaration* refers to indicator values that are described in string format. Suitable indicators include qualitative indicators such as descriptions of service level agreements.
- BooleanIndicatorDeclaration. A *BooleanIndicatorDeclaration* refers to indicator values that are described in Boolean format, that is, indicators having binary values assigned (e.g., yes/no). For example, such indicators refer to the question of whether a certain technology is used within an organization.
- DecimalIndicatorDeclaration. A *DecimalIndicatorDeclaration* refers to indicator values that are described in decimal format. It represents, for example, quantitative performance indicators, such as the number of workplaces, as well as cost indicators.

One of the most important relations within the concepts described above is the relation between the Benchmark and its associated participation and the involved organizations. The Participation concept is only required to model the ternary relation between a benchmark, its participants and their provided values. This, however, comes at the cost of a more complicated ontology usage, as this intermediate concept has to be considered for related queries. Moreover, using DUL, one would usually expect that for participation relations, a *DUL:involvesAgent* and/or its inverse *DUL:isAgentInvolvedIn* is specified. Unfortunately, the Nary Participation pattern does not include statements to establish such a relation. This issue is addressed by utilizing complex role inclusion [29]. Thus, to define the original *DUL:involvesAgent* property (which also implies its inverse), a property chain consisting of the inverse of *hasBenchmark* ($hasBM^{-1}$) and the *hasOrganization* (*hasOrg*) property has been specified to imply the *DUL:involvesAgent* property and is formally represented as

$$\begin{aligned} hasBM^{-1} \circ hasOrg \sqsubseteq involvesAgent. \end{aligned}$$

As mentioned before, indicators of a specific benchmark (i.e., their instantiation) are linked to a single category using the *categorizesIndicator* object property. If, for example, category *A* nests category *B*, which already nests category *(*C), category *(*A) also nests category *(*C) and is referred to as a transitive relation of categories. This transitiveness does not apply to indicators linked by *categorizesIndicator*. To ensure that category *A* also includes all indicators that are categorized by one of its sub-categories, the following needs to be introduced:

$$\begin{aligned} hasChild \circ categorizesIndicator\sqsubseteq categorizesIndictor \end{aligned}$$

**Fig. 3:**
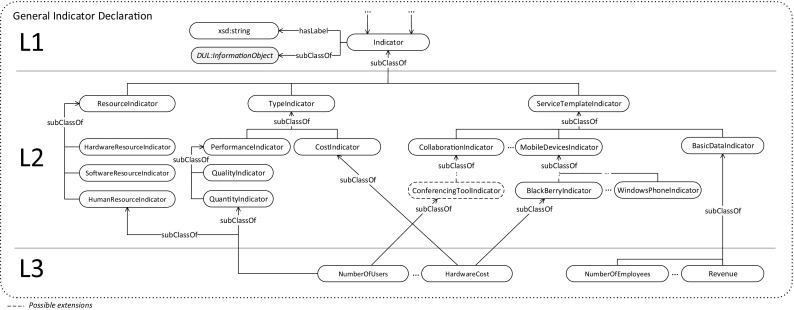
General Indicator Declaration including exemplary indicators. Solid arrows indicate taxonomic relationships, and concepts with dashed borders indicate examples of more fine-grained extensions of the service template. Statements of disjoint classes are omitted to improve readability

### General Indicator Declaration

General Indicator Declaration section (cf. Fig. 3) introduces a taxonomic description of the indicators used in IT benchmarks. This starts from the top level with the general Indicator class and moves on to the more specific concept of an indicator (for example, the MobileDevicesIndicator in Fig. 3) that refers to indicators that are instantiated by an individual benchmark. In other words, instances of indicators form the entities that are linked to a benchmark structure described in Sect. 4.1.1. The most specific classes, which contain the subset of indicator instances, refer to the same (specific) indicator, as they are included in different individual benchmarks.

The taxonomy is implemented in three different layers (L1 to L3). Except for the first layer, layers 2 and 3 consist of a large set of classes partitioning the set of available indicators by different characteristics using *subClassOf* definitions. Due to the large number of indicators, in the following, we refer to a complete layer, rather than to a single concept, to provide a more coarse-grained description instead of describing each concept individually.

- L1: General Concept. The top layer of the taxonomy only consists of the root concept of the taxonomy: the *Indicator* class. This class constitutes the set of all instantiated indicators and is grounded in the *DUL:InformationObject* class to describe more abstract pieces of information to be realized by a questionnaire. Furthermore, the elementary data property *hasLabel* is defined and used by indicator instances to specify at least one label used as an indicator name within a specific benchmark (equivalent to an individual benchmark).
- L2: Indicator Dimensions. Indicators may be classified using different dimensions. In the current ontology version, we introduced dimensions for (i) the (IT) service that is measured according to a service template for the structure of an IT service based on recent research activities [46, 47], (ii) the specific type of questions to which an indicator is assigned (i.e., whether it is a cost or performance indicator) and (iii) the type of resource (i.e., hardware, software, or human resource) to which the indicator refers. There is no natural order for performing hierarchical splits among the different dimensions; thus, all possible splits are performed in parallel in the intermediate layer of the taxonomy. One dimension subdivides the set of all indicators into smaller (sub-)sets. These subsets of indicators belong to a certain service or a certain type of indicator. Concepts within the same dimension and the same hierarchical level are pairwise disjoint. Specifically, an indicator (L3) may only be of one type for each dimension. Moreover, except for the service template dimension, a dimension does not necessarily need to cover all indicators. Thus, it is possible to specify indicators that are neither cost nor performance indicators and/or do not imply a resource type. Indicators belonging to the basic data service template (represented by the BasicDataIndicator class) describe the core data of participating organizations (e.g., the yearly revenue), the number of employees, and structural information about the organization among others. Most indicators are neither performance nor cost indicators and therefore are separated in this basic data service. The remaining services refer to more specific IT services, such as those regarding user collaboration or IT infrastructure. The resource dimension refers to the resources described by a specific indicator. Possible resources include hardware, software and human resources. Performance indicators may be further split into quality (e.g., referring to service level agreements) and quantity indicators. There are, however, performance indicators that are neither quality nor quantity indicators. Dimensions can have their own intrinsic hierarchy, describing the different concepts they consist of in different granularities. For example, as shown in Fig. 3, the collaboration indicators are additionally specified by the ConferencingToolIndicator class in the service template dimension. Another example at a more specific level includes indicators to be further split according to different quality or hardware standards that they describe, such as BlackBerry or WindowsPhones within the MobileDevices service template. In contrast to the introduced intermediate abstraction levels shown in Fig. 3, the current implementation of the ontology contains two levels of abstraction within the service template dimension. (Additional splits are marked as possible extensions.) The first abstraction refers to the service name, and the second abstraction refers to an additional sub-classification, for example as, currently implemented for the MobileDevices service. In contrast to the service template dimension, descriptions of other dimensions are expected to remain more constant.
- L3: Indicators and Relationships. The bottom layer of the indicator taxonomy consists of the most specific indicator descriptions, referring to a single indicator instantiated by individual benchmarks rather than to an indicator categorization. As explained above, such indicators are classified in one or multiple dimensions (using *subClassOf* definitions) but are only covered completely within the service template dimension.

### Ontology Summary

At present, the IT benchmarking ontology consists of a number of statements, which are summarized in Table 3. The number of classes corresponds to the concepts described in the previous sections, including the 20 top-level service classes (one of which is the basic data service), corresponding to IT services that are commonly measured within an IT benchmark, and the 1064 L3 indicator classes, corresponding to key performance indicators that are measured during an IT benchmark. Entities of the indicator taxonomy do not have their own properties defined but rather inherit the *hasLabel* property from their *Indicator* base class. Therefore, only a small set of object and data properties need to be additionally defined, and they are shown in Fig. 2. Currently, the majority of axioms refer to the number of *SubClassOf* definitions. However, axioms on the domain and range of object properties and statements relevant to the characterization of disjoint classes also exist. The number of annotations includes bilingual (viz. English and German) *rdfs:label* and *rdfs:comment* for all classes. The description logic expressiveness for the benchmarking ontology is $\mathcal {SRIQ(D)}$.

**Table 3:** Number of classes, properties, axioms and annotations in the ITBM ontology

Ontology Metric	#	Ontology Metric	#
Classes	1192	Logical Axioms	3287
Object Properties	123	Annotations	5264
Data Properties	9		

## Application and Use Case of the ITBM Ontology

### System Architecture

**Fig. 4:**
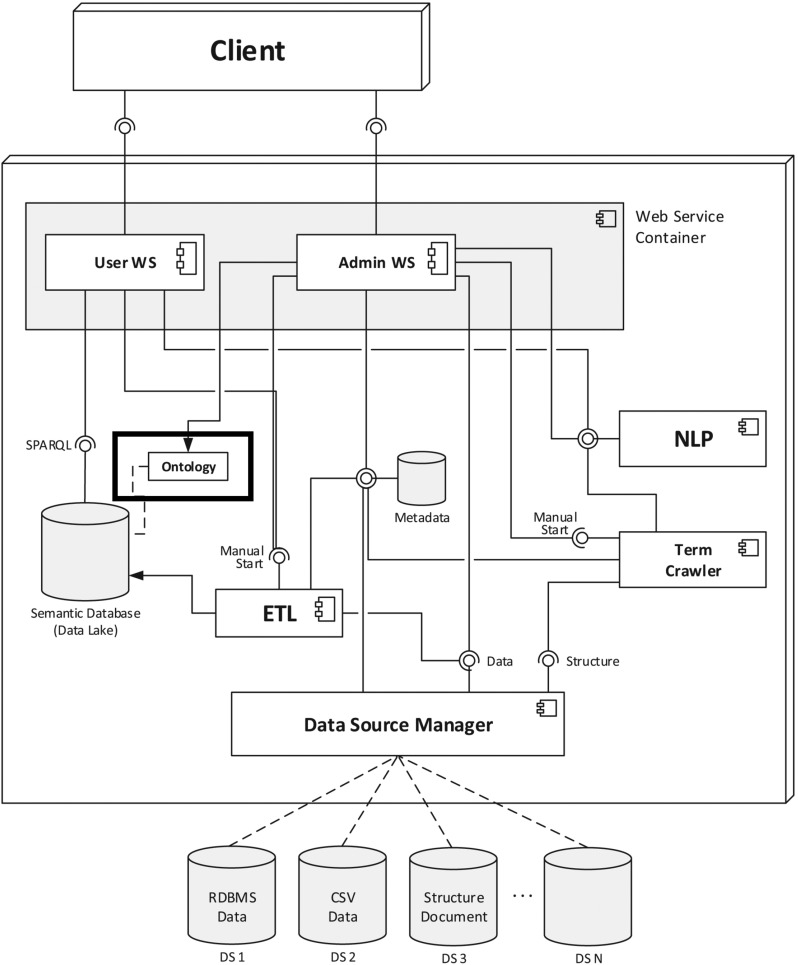
System architecture for ontology-based data integration [43]

Because the ITBM ontology is built for the purpose of data access in the domain of IT benchmarking and is based on research activities on strategic and service-oriented IT benchmarking initiatives, the application of the ITBM ontology within a web-based system architecture for data access will be described as follows. The main focus of the presented prototype is on (i) accessing data from external databases through the use of natural language queries and (ii) supporting the (semi-)automatic mapping of concepts of the ontology with data points of the attached databases. The complete system architecture is described in more detail in [43]. Figure 4 illustrates the complete system architecture. A black border highlights the implementation of the ontology within the system.

The connection of external data sources is configured through the use of the data source manager. The data source manager ensures the correct mapping of the relational structure of the attached databases to the corresponding ontology by detecting changes in the relational scheme. These changes are reflected in a new version number for the data source.

The Extract Transform Load (ETL) module is implemented for the data integration task (see below). This process is based on a twofold mapping of the metadata stored in the metadata repository. The first part (part 1) specifies a set of transformation rules to transform external data models (i.e., a database scheme) into a virtual model, where each virtual table (i.e., SQL queries, referred to as *Generators*) corresponds to an ontological concept. The specification in the second part (part 2) utilizes this virtual model to map table instances (i.e., rows) to instances of the corresponding concepts. Examples of those metadata are provided in Listing 1. A *generator* created on top of the organization table of an external database is specified (part 1) and mapped to the *DUL:Organization* concept of the ontology (part 2). To keep the example simple, both further transformations (e.g., filters) and specifications of links to other generators (i.e., foreign keys) have been omitted.
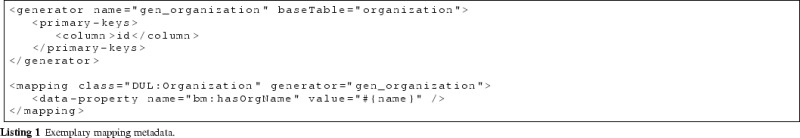


Creating such mappings for all tables/concepts is a tedious process; thus, to support the mapping of database contents to ontology concepts (i.e., creating the second part of metadata), a (semi-)automatic mapping recommender is implemented. Here, ”(semi-)automatic” refers to the fact that mappings are initially recommended by the system but not applied automatically so that human interaction is needed to confirm recommended mappings for the purpose of quality assurance. The system supports two different types of mapping recommendations. The first type of recommendation assumes that a whole database table corresponds to an existing ontology concept and the second type of recommendation that each database table record is mapped to a different ontology concept. Additional details are provided as follows:

- *Mapping (virtual) tables to ontology concepts:* Often, a (physical) table from the original database schema directly corresponds to a concept defined in the ontology. In this case, all records of this table are converted to instances of this concept. Note that if concepts in the ontology are specified on a more fine- or coarse-grained level of abstraction, such a table may still be constructed virtually using appropriate SQL statements (e.g., JOINs); within the scope of the system, this type of table has been referred to as *Generators*. For example, consider the database table *”organization”*, which contains all the organization names of the participants for a specific benchmark. Thus, the rows of this table directly reflect the instances of the *DUL:Organization* concept that need to be integrated. The matching of database table names to the concepts of the ontology is based on different similarity metrics. This mapping is realized by the mapping recommender. For quality assurance, the mapping candidates are presented to the user for confirmation. An example of such a mapping is given in Listing 1. In this example, the mapping process for two organizations, named *Organization 1* and *Organization 2* (cf. the name column of the organization table), results in the corresponding triples, which are shown in the following Listing 2. 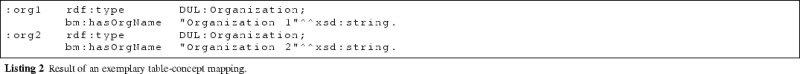

- *Mapping (virtual) table records to ontology concepts:* Sometimes records are not meant to be converted to instances of the same concept but rather are partitioned to different concepts. In this case, a specific table is chosen, and each of its records is converted to one instance of a specific concept of the ontology. For example, a database table of *indicators* may consist of the different indicators that are captured during the benchmark. In this case, however, each row of the table corresponds to an individual concept within the ontology. Consequently, the mapping recommender searches for a corresponding concept for each row of the table within the ontology by applying similarity metrics to each of these rows/concepts. As a result, a mapping entry is generated for every table row. Listing 3 shows the mapping results for the *NumberOfEmployees* table and the *Revenue* table (cf. Fig. 3) labeled with *Number of employees* and *Yearly revenue* to their corresponding ontology concepts.

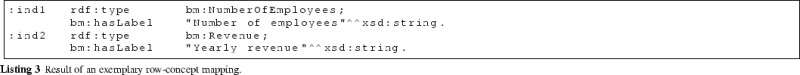

**Fig. 5:**
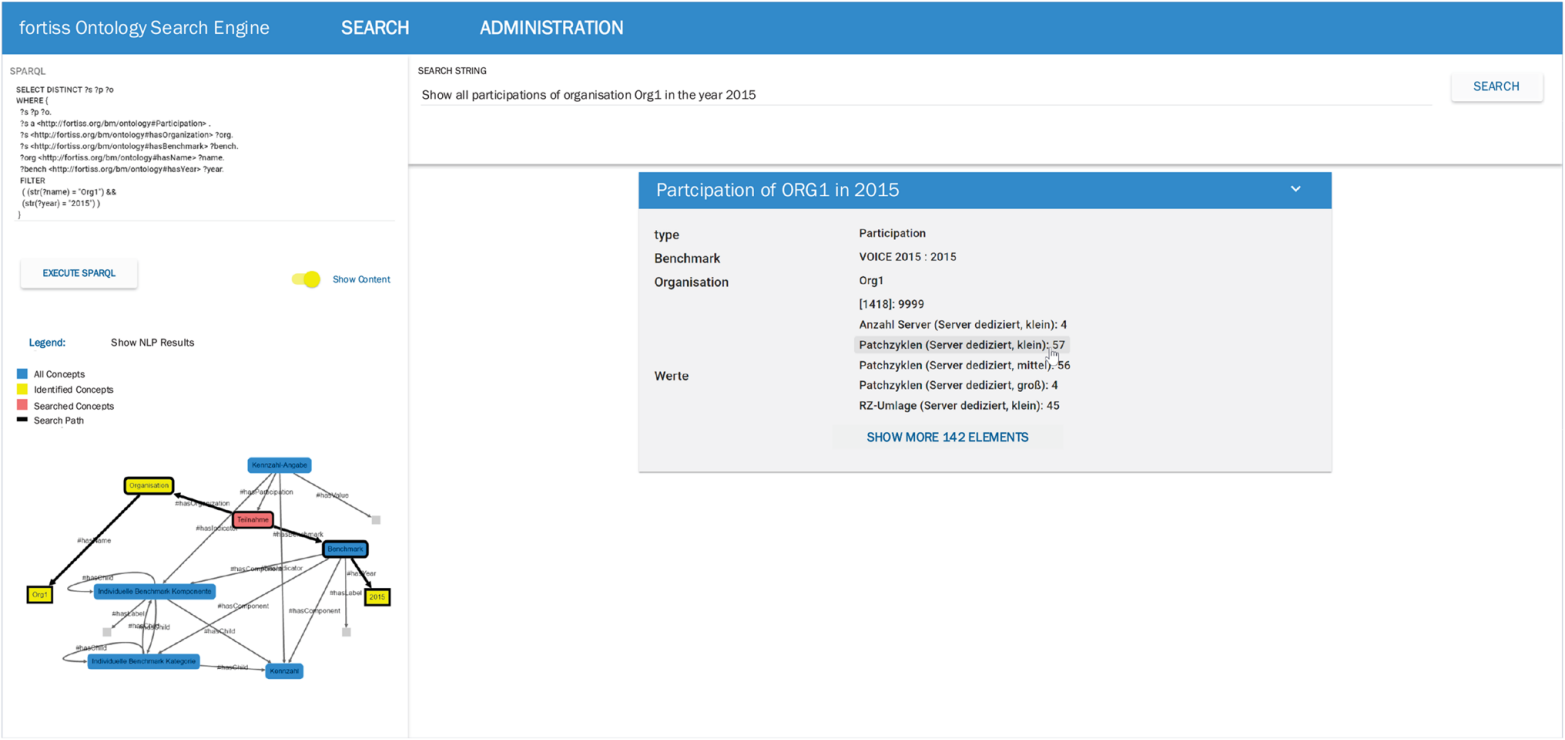
Client-side search mask for ontology-based data access in ITBM (based on Pfaff and Krcmar [43])

Both of these mapping cases are implemented through the use of the same underlying bipartite matching algorithm (based on Kuhn and Yaw [31]) differing from its run-time configuration. In the first case (i.e., mapping (virtual) tables to ontology concepts), the total set of virtual and physical table names and the names of the ontology concepts are used as input configuration. In the second case (i.e., mapping (virtual) table records to ontology concepts), the total set of rows of a specified table and the names of ontology concepts are used as the input configuration for the mapping algorithm.

These mappings represent the assignment between the entities and attributes from the data sources and their corresponding concepts and properties of the ontology. According to these mappings, the data integration process is stepwise performed as follows (executed by the extract, transform, load (ETL) module):

- Load the mapping entries in accordance with the selected versions of both the ontology and the connected databases.
- Apply transformation rules to the relational models of the connected databases to create an intermediate model with bidirectional links between tables; this is realized by creating a set of SQL statements wrapped around the original tables.
- Load data from attached databases via the data source manager using the generated SQL statements.
- According to the second part of the mapping specifications, map tables to concepts by converting their rows into instances of the ontology using the triple-store format.
- Load the data into the semantic database as a new graph within the semantic database; old data are kept in the old graph.
- Check whether the new graph differs from the data loaded in previous ETL iterations and log changes.

A web interface can be used to access the attached data sources via natural language text (text-to-sparql). This client-side user interface is implemented using AngularJS [20] and is shown in Fig. 5. As a result of most of the data sets being in German, the output of the user query (”Show all participations of organisation Org1 in the year 2015”) is presented in the German language. Directly underneath the automatically generated SPARQL query, the search tree within the ontology is presented. Blue nodes represent the corresponding concepts in the ontology when the user searches for data sets. In addition, the automatically generated SPARQL queries can be directly edited or reformulated using the web interface.

### Competency Questions and SPARQL Queries

Because data access is generally performed through the use of natural language queries (see Sect. 5) and can also be performed by executing SPARQL queries, the correspondence between the CQs and the resulting SPARQL queries is outlined in the following, focusing on the most complex or interesting queries (see Tables 4 and 5).

**Table 4:** Excerpt of competency questions and corresponding SPARQL queries for indicator structure and individual benchmarks

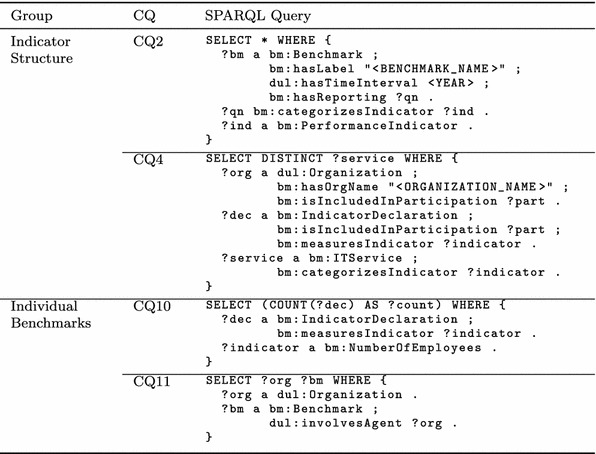	

CQ2 asks for all performance indicators that have been collected in a specific benchmark of a specific year. In SPARQL, these performance indicators are queried by filtering the set of all benchmarks in accordance with the defined benchmark name and year. As previously stated, all indicators of a specific benchmark are linked to a specific questionnaire (see Sect. 4.1.1). Thus, all performance indicators that are linked to this questionnaire are queried. Please note that the root questionnaire directly categorizes all indicators linked to a benchmark due to the *bm:categorizesIndicator* property chain (see Sect. 4.1.2).

CQ4 asks for the existence of all IT services to which an organization responded within a specific benchmark (i.e., values for indicators are provided by the organization). An organization can participate within various benchmarks; therefore, all its participations, the corresponding indicator declarations and its indicators are queried. As a result of this CQ the result set of this query only contains indicators that have been specified within a specific IT service.

CQ10 asks for the total number of responses provided by an organization for the specific indicator *bm:NumberOf* *Employees*. The resulting SPARQL counts the number of indicator declaration instances referring to this indicator.

Next, CQ11 queries all participations of all organizations and the benchmarks they participated in using the introduced object property chain, which infers the *dul:involvesAgent* property for all benchmarks and organizations.

**Table 5:** Excerpt of competency questions and corresponding SPARQL queries for participants and values

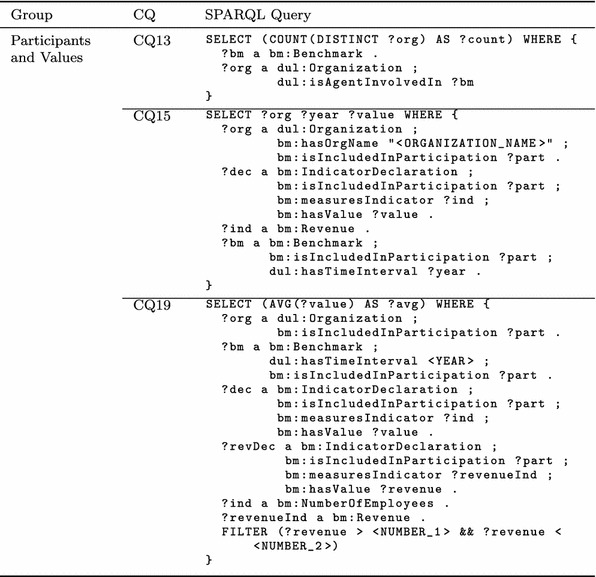	

CQ13 queries the number of organizations that participated in at least one benchmark. Similar to CQ11, this is achieved using the inverse of *dul:involvesAgent*, that is, *dul:isAgentInvolvedIn*, and then by counting over the distinct result set. Note that without using the DISTINCT command, organizations that have participated in more than one benchmark would be counted multiple times.

By CQ15, the yearly revenue of a specific organization is queried. Using the abstract property *bm:isIncludedIn* *Participation*, the organization is identified by its name, the years are queried using the specific benchmarks that the organization participated in, and the corresponding values of the revenues are returned.

By CQ19, the average number of employees of all organizations in a specified year with a revenue within a specified range is calculated. Again, the abstract *bm:isIncludedIn* *Participation* property is used to query the participation pattern. Thus, the organizations, the benchmarks, the indicator declaration of the revenue, and the indicator declaration of the number of employees are queried. The resulting set of values is filtered to match the specified revenue range and the number of employees is averaged and returned.

## Conclusion and Outlook

This work introduces a domain-specific ontology for the domain of IT benchmarking to bridge the gap between a systematic characterization of IT services, which is closely related to ITSM, and their data-based valuation in the context of IT benchmarking. This ontology will serve as a universal link for the semantic integration of different types of different benchmarking data. It is based on ITBM data and IT service catalogs collected over the last eight years in the context of research activities at fortiss and TUM. The ontology is implemented in an evaluation and reporting tool for ITBM as a core concept for the data access and connection of different ITBM data sources.

The layered indicator structure addresses two major aspects that have to be considered when developing an ontology for IT benchmarking. First, it provides the flexibility needed when assembling a new service based on individual indicators, as it separates the service structure from the indicator structure. Second, new indicators can be introduced or modified apart from the service structure. This eases the maintenance of the ontology for future improvements and customizations on both sides; the indicators and the service structure.

At present, the ontology is divided into three sections: (i) Individual Benchmarks, (ii) Participants and Values, and (iii) General Indicator Declaration. Therefore, a separation of the general time-related information of a benchmark and the structural information of the utilized questionnaires from the corresponding data that are connected to a specific indicator is achieved. For future work, *General Indicator Declaration* section, which is implemented in a three-layer (L1 to L3) architecture that considers the relevant relations and dependencies of all indicators within a benchmark could be extended by introducing further categorization within the service template dimension as well as by introducing a new dimension, therein consisting of a set of several disjoint L2 classes in the L2 layer referring to different unit types. It could be the case that various indicators share their unit or may be of different indicator unit types within different IT services. For example, one performance indicator can be represented by a single number (e.g., number of physical hosts), whereas another indicator can be indicated by textual values (e.g., the name of a specific software product). The same holds true for cost indicators, which might be expressed in different currencies (e.g., Euros or Dollars) or other units (e.g., full time equivalents (FTEs)). In addition, some indicators that are neither cost nor performance indicators (i.e., that are not classified within this dimension) could also share their type of unit with cost or performance indicators. For example, the yearly revenue, which is part of the basic data service, could be seen as a shared cost indicator, and the number of employees of an organization can be an example of a shared performance indicator. To overcome this fragmentation of different indicator types, the dimension of the *General Indicator Declaration* could facilitate defining a set of restrictions across different dimensions, i.e., classes referring to unit types could be declared pairwise disjoint from classes belonging to different dimensions (e.g., CostIndicators could be defined disjoint from any type of textual unit types). By directly assigning the unit type to an indicator, a more fine-grained indicator categorization would be achieved.

The ITBM ontology is already implemented as bilingual (viz. English and German) using annotation properties, and the application that the ontology is part of handles terminological transformations through the NLP module, which is sufficient for the current use case, as all concepts of the ontology are already lemmatized. In the future, this linguistic information could be further improved through the use of an ontology lexicon such as the lexicon model for ontologies (lemon) as introduced by Cimian et al. [7]. In this manner, it could be possible to improve the results of the NLP module, especially if the ITBM ontology is continuously expanding and if multiple languages and vocabularies need to be associated with the ontology.
